# Discovering the structure and organization of a free Cantonese emotion-label word association graph to understand mental lexicons of emotions

**DOI:** 10.1038/s41598-022-23995-z

**Published:** 2022-11-15

**Authors:** Ting Yat Wong, Zhiqian Fang, Yat To Yu, Charlton Cheung, Christy L. M. Hui, Brita Elvevåg, Simon De Deyne, Pak Chung Sham, Eric Y. H. Chen

**Affiliations:** 1 Department of Psychiatry, School of Clinical Medicine, Li Ka Shing Faculty of Medicine, University of Hong Kong, Hong Kong, China; 2 Neurodevelopment and Psychosis Section, Department of Psychiatry, Perelman School of Medicine, University of Pennsylvania, Philadelphia, USA; 3 Department of Clinical Medicine, University of Tromsø—The Arctic University of Norway, Tromsø, Norway; 4 School of Psychological Sciences, University of Melbourne, Melbourne, Australia; 5 State Key Laboratory of Brain and Cognitive Sciences, University of Hong Kong, Hong Kong, China

**Keywords:** Psychology, Human behaviour

## Abstract

Emotions are not necessarily universal across different languages and cultures. Mental lexicons of emotions depend strongly on contextual factors, such as language and culture. The Chinese language has unique linguistic properties that are different from other languages. As a main variant of Chinese, Cantonese has some emotional expressions that are only used by Cantonese speakers. Previous work on Chinese emotional vocabularies focused primarily on Mandarin. However, little is known about Cantonese emotion vocabularies. This is important since both language variants might have distinct emotional expressions, despite sharing the same writing system. To explore the structure and organization of Cantonese-label emotion words, we selected 79 highly representative emotion cue words from an ongoing large-scale Cantonese word association study (SWOW-HK). We aimed to identify the categories of these emotion words and non-emotion words that related to emotion concepts. Hierarchical cluster analysis was used to generate word clusters and investigate the underlying emotion dimensions. As the cluster quality was low in hierarchical clustering, we further constructed an emotion graph using a network approach to explore how emotions are organized in the Cantonese mental lexicon. With the support of emotion knowledge, the emotion graph defined more distinct emotion categories. The identified network communities covered basic emotions such as love, happiness, and sadness. Our results demonstrate that mental lexicon graphs constructed from free associations of Cantonese emotion-label words can reveal fine categories of emotions and their relevant concepts.

## Introduction

Emotions are not necessarily universal across different languages and cultures^1^. Many contemporary psychological models of emotion view it as a specific and hardwired domain that is distinct from other mental processes such as cognition^2–6^. This might suggest that language plays no role in the acquisition of emotion concepts. However, a growing body of literature has suggested that language can shape our emotional experiences and meaning^7,8^. A recent study has examined 2474 spoken languages and proposed that language can shape uniqueness in emotional meaning and experiences^9^. From a neurobiological perspective, an overlapping pattern of brain regions implicated in both semantic processing and experiences of discrete emotions suggests a role of language in emotion^10^. Accordingly, psychological constructionist perspectives proposed that language is connected with conceptual knowledge and alters the construction of emotional perception^11^. In the Conceptual Act Theory (CAT) of emotion, the compound “emotion” is contributed by three elements: affect, exteroceptive sensations, and concept knowledge^5^. Affect and exteroceptive sensations are only meaningful when linked to instances of emotion categories^12^.

Information about words represented in the mental lexicon is not limited to meaning but includes other information as well such as phonation, and syntactic features^13,14^. The emotion concepts, emotional experiences, and meaning could form the mental representation of emotional words stored in memory, namely the mental lexicon of emotion. Mental lexicons reflect the shared subjective meaning, shaped by our experiences, and the linguistic environment^15^. Therefore, mental lexicons of emotions depend strongly on contextual factors, such as language and culture. Researchers have studied the lexicon of emotional words and how emotional experiences are organized or structured in our minds in different human languages. Studies on English mental lexicons of emotions have revealed the hierarchical structure of different emotional words using a prototype approach^16^. With the same hierarchical approach, other languages such as Basque and Indonesian were explored as well^17,18^. Comparison of emotion lexicons in different languages has also led to a deeper understanding of the basic-level emotions and other superordinate emotions across cultures. For example, shame is one of the superordinate emotions under the *sadness* cluster in Indonesian^17^ while it is located under a *fear* cluster in Dutch^19^. Yet in Chinese^20^ and Japanese^21^, shame would form a separate emotion cluster. The different clustering memberships of the same emotional word indicated that the conceptual understanding of emotions may vary across languages and cultures^17^.

The Chinese language has unique linguistic properties that are different from other languages (e.g., English). Chinese characters have no unitary definition and thus are rarely used in isolation^22^. In general, Chinese words are composed of two or more individual characters, leading to an abstract term. For instance, the character 驚 (ging1) means *fear* but it could also be combined with 訝 (ngaa6) or 喜 (hei2) to express *surprise*. Intuitively, the word 驚訝 (*surprise*, ging1ngaa6) represents a negative feeling of *surprise* while the word 驚喜 (*surprise*, ging1hei2) conveys a positive one. Thus, Chinese text has a compositionally richer semantic meaning^23^. Though many studies were conducted on Chinese emotion words, most research on Chinese emotional vocabularies focused on Mandarin but not Cantonese^22,24–27^. There are more than 80 million people are using Cantonese as their first language^28^. Although Cantonese and Mandarin share the same base writing system, the two are quite different when spoken and sometimes, they have their distinctiveness in emotional expression. For example, only available in Cantonese, the word 陰功 (*pathetic*, jam1gung1) literally means "merits from the netherworld” in Cantonese and it is usually used as a metaphor to express a feeling of pity and sadness. Since language is a carrier of culture^29,30^, the emotion semantics of Cantonese and Mandarin words could potentially vary.

In the past decades, network models have been developed and widely applied in understanding the organization of the mental lexicon. Small-world scale-free complex network, one of the major types of network model, enables us to explore the organizational features of the mental lexicon at different levels, including macroscopic, mesoscopic, and microscopic levels^31^. Macroscopic properties characterize the global organization, mesoscopic properties describe the subsets of nodes and word meanings, and microscopic properties focus on the connections of a single node to the rest of the network^15^. With this approach, we can investigate multiple levels of mental lexicon simultaneously to deepen our understanding of the categories of Cantonese emotion lexicons as well the emotional characteristics of unique Cantonese lexicons and their neighbor nodes.

With advances in network science, the current study aims to explore the structure of the Cantonese mental lexicon of emotions. As part of a larger study on semantic association as elicited using the continued word association task (WAT) in a Cantonese-speaking community sample in Hong Kong, we aim to understand these Cantonese emotion-label words and their related concepts. Specifically, this study aims to:

1. Generate clusters of emotions given the affective properties of these Cantonese emotion cue words (i.e., valence, arousal, dominance, and concreteness). Based on the previous studies described above^17,19,21^, four to six clusters of emotions were found in other languages (i.e., basic emotion prototypes such as fear and anger). Are emotion words organized similarly in Cantonese?
2. Build an emotion mental lexicon network with both emotion words and emotion-related to examine the mesoscopic and microscopic properties of a contextualized (e.g., including events, agents, objects, and other aspects of meaning) emotion network. At the mesoscopic level, community detection was conducted to explore different emotion clusters that are present in the network. At the microscopic level, we inspected pairs of synonymic emotion words (e.g., *surprise,* ging1hei2 驚喜 vs. ging1 ngaa6 驚訝) and how they relate to other nodes. These provide a structural explanation for the different affective properties of these synonymic emotion words.

## Methods

### Participants

Healthy participants (*n* = 10,693) in the community were recruited via the university mass email, university campus events, as well as social media platforms such as Facebook. Eligible participants were aged 18–60 years and were Cantonese-speaking Chinese who identified themselves as fluent Chinese writers and readers. This study was approved by the Institutional Review Board of the University of Hong Kong/Hospital Authority Hong Kong West Cluster (UW14-167). The authors assert that all procedures contributing to this work comply with the ethical standards of IRB on human experimentation and with the Helsinki Declaration. Electronic informed consent to participate has been obtained from all participants.

### Word Association Task (WAT)

Previous findings suggested that internal language models derived from word association data substantially outperformed word-embedding models based on external text corpus data^32^. Our model for the current study was based on data collected from work association tasks. Participants were invited to participate in an online study through our website https://smallworldofwords.org/hk/. Basic demographic information was required, including age, gender, education level, whether they are native Cantonese speakers, their Cantonese dialects (Hong Kong, Macau, Guangzhou, others), and whether they were on any antipsychotic medication.

The word association task (WAT)^15,33^ was then administered. In this task, 20 cue words were displayed on the screen one by one. These cue words were one to three-character Cantonese words that have been randomly selected from a previously compiled list, more details are included in the “Cantonese cue word list generation” section. Participants were instructed to think of words (nouns, verbs, and/or adjectives) related to each of the cue words, and to enter the first three unique associated words that came across their minds. For example, the cue word 交通 (*transport*) can be associated with 巴士 (*bus*), 駕車 (*drive*), and 方便 (*convenient*). If participants did not understand a particular cue word or if no additional association could be recalled, they could skip to the next cue. Participants were also instructed to give associations to the cue words only and avoid associations related to their previous responses. Phrases and full sentences were discouraged.

#### Cantonese cue word list generation

To compile a comprehensive and representative list of commonly used Cantonese words for the WAT, a three-step word selection process has been followed.

First, 1058 2-character cue words containing nouns, verbs, adjectives, and adverbs were selected based on familiarity from two major Chinese corpora, namely the Ghent University SUBTLEX Mandarin word frequency list^34^ and the Linguistics Corpus of Mid-20th Century Hong Kong Cantonese^35^.

Second, we ran a pilot online WAT with self-reported healthy participants to gather additional cue words from their responses. Participants responded to each of the cue words with at most 3 associations. After receiving 20 responses per cue word, the most frequent answers were clustered according to content, from all of which 1324 additional cue words were uncovered and added to the cue word list. We excluded those cue words with more than 50% of “unknown” responses (i.e., where participants did not know the cue word or could not think of associations).

Third, 38 cue words that are commonly used by Cantonese-speaking patients with psychosis, such as those used to describe symptoms, were taken from interviews and assessments and added to the list. All psychopathology-related cue words were used by more than one patient. Therefore, the final cue word list consists of 2420 common Cantonese words.

#### Emotionality and properties of cue words

An independent group of 100 healthy participants aged between 18 and 60 years was recruited through convenience sampling in Hong Kong to rate the emotional properties of these 2420 cue words in Microsoft Excel. All participants were able to read and understand Cantonese, had access to a computer and were able to use Microsoft Excel software. Additionally, they were not currently under antipsychotic medication and did not have any history of psychiatric disorders. Ethics approval was obtained, and all participants provided written informed consent to participate in this rating study.

Emotional properties included (1) valence, (2) arousal, (3) dominance, and (4) concreteness. The first three properties measure the subjective feelings associated with the words on a 9-point Likert scale^36^. Valence measures the degree of which a word invokes positive or negative emotions, with lower scores indicating more negative emotions, and vice versa. Arousal measures the degree of which the word invokes feelings from calmness (lower scores) to excitement (higher scores). Dominance measures the degree of which the word invokes feelings of passivity and loss of control (lower scores) and feelings of dominance and control (higher scores). Concreteness is rated on a 5-point scale^37^, with 1 indicating more abstractness and 5 indicating more concreteness. Concrete words are defined as words that were learned primarily from experience or sensory input, whereas abstract words are defined as words learned primarily from language or using other words.

Each participant was asked to complete one of the four scales, with each scale taking approximately 2.5 hours. Participants completed the task using their computer in their own time and were asked to return the completed survey within 1 week after study entry. Upon completing the ratings, each participant received compensation of HKD $300. Demographic information including age, gender, and years of education was also collected prior to the ratings.

### Data preprocessing and analyses

#### Word association data preprocessing

Several preprocessing steps were taken to the data. First, we removed spaces, empty sets, special characters, and non-Chinese responses. Responses with more than six Chinese characters were excluded. Second, within a participant, only the first three unique responses were included while duplicated responses were recoded as “NA”. Then, in the cues list, we combined cues that are synonyms without much difference in terms of their meaning (e.g., 唔開心, 不開心; both 唔 (m4) and 不 (bat1) means no, where 開心 (hoi1sam1) means *happy*. Thus, these two words together mean *unhappiness*). The emotion words used for the current study are limited to the basic emotion types. Out of the full list, 79 Cantonese emotion cue words were identified based on a highly representative Chinese emotion word list^38^ (see Table 1 for the complete list and Table S1 in the supplementary material for their emotional ratings by independent raters).

**Table 1:** Chinese and translation of 79 Cantonese emotion words.

Chinese	Translation	Chinese	Translation	Chinese	Translation	Chinese	Translation
友情 jau5 cing5	Friendship	開心 hoi1 sam1	Happiness	難過 naan4 gwo3	Upset	嫌棄 jim4 hei3	Despise
喜悅 hei2 jyut6	Joy	驚喜 ging1 hei2	Surprise	驕傲 giu1 ngou6	Proud	害怕 hoi6 paa3	Scared
喜愛 hei2 oi3	Favorite	高興 gou1 hing1	Happy	驚慌 ging1 fong1	Frightened	心痛 sam1 tung3	Heartbroken
喜歡 hei2 fun1	Like	乞人憎 hat1 jan4 zang1	Loathsome	驚訝 ging1 ngaa6	Surprise	憂慮 jau1 leoi6	Worry
快樂 faai3 lok6	Happiness	傲慢 ngou6 maan6	Arrogant	不喜歡 bat1 hei2 fun1	Dislike	憤怒 fan5 nou6	Angry
愉快 jyu4 faai3	Happy	可悲 ho2 bei1	Pathetic	不滿 bat1 mun5	Dissatisfied	擔心 daam1 sam1	Worry
愛 oi3	Love	害羞 hoi6 sau1	Shy	仇恨 sau4 han6	Hatred	暴躁 bou6 cou3	Irritable
戀愛 lyun2 oi3	Love	失落 sat1 lok6	Down	傷心 soeng1 sam1	Sad	氣憤 hei3 fan5	Angry
放鬆 fong3 sung1	Relaxed	恐懼 hung2 geoi6	Fear	冷漠 laang5 mok6	Indifferent	沮喪 zeoi2 song3	Frustrated
歡喜 fun1 hei2	Joy	悲傷 bei1 soeng1	Sadness	內疚 noi6 gau3	Guilty	焦慮 ziu1 leoi6	Anxious
歡樂 fun1 lok6	Joy	悲哀 bei1 oi1	Sorrow	厭惡 jim3 wu3	Disgusted	煩 faan4	Annoyed
滿足 mun5 zuk1	Satisfied	悲慘 bei1 caam2	Miserable	可怕 ho2 paa3	Terrified	生氣 sang1 hei3	Angry
激情 gik1 cing4	Passion	憎恨 zang1 han6	Hatred	可憐 ho2 lin4	Miserable	絕望 zyut6 mong6	Despair
興奮 hing1 fan5	Excited	擔憂 daam1 jau1	Worry	哀傷 oi1 soeng1	Sad	緊張 gan2 zoeng1	Nervous
舒暢 syu1 coeng3	Comfortable	激動 gik1 dung6	Excited	唔開心 m4 hoi1 sam1	Sad	討厭 tou2 jim3	Dislike
舒服 syu1 fuk6	Comfortable	煩厭 faan4 jim3	Boredom	嘔心 au2 sam1	Disgusted	難受 naan4 sau6	Unhappy
舒適 syu1 sik1	Comfortable	痛心 tung3 sam1	Agonized	困擾 kwan3 jiu5	Distressed	驚 geng1	Fear
親情 can1 cing4	Family affection	痛苦 tung3 fu2	Agony	好煩 hou2 faan4	Very annoying	驚嚇 ging1 haak3	Startled
輕鬆 hing1 sung1	Relaxed	苦悶 fu2 mun6	Bored	妒忌 dou3 gei6	Jealous	驚恐 ging1 hung2	Panicked
鍾意 zung1 ji3	Like	陰功 jam1 gung1	Pathetic	嫉妒 zat6 dou3	Jealousy		

#### Dimensions of emotion-label words

Hierarchical clustering is widely used in examining structures and dimensions of emotion lexicons in a variety of languages^16–18,27^. To obtain comparable clusters, we adopted a similar approach. Hierarchical clustering on emotion words was applied based on a matrix in which each word was represented by a vector consisting of valence, arousal, dominance, and concreteness scalars. Silhouette coefficients were used to determine the optimal number of clusters. Further, the stability of the optimal solution was assessed by calculating silhouette coefficients from two to ten cluster solutions of each combination of distance (i.e., Manhattan, Euclidean) and linkage function (i.e., average, complete, single, ward). To understand the clustering rules from the data-driven results, we compared the differences between obtained clusters by visualizing the mean ratings of valence, arousal, dominance, and concreteness of emotion word cues.

#### Association network construction

First, a word association network (*G*_R123_) using all cues (*N* = 2420) was constructed from a weighted adjacency matrix where both the rows and columns correspond to the different cues and contain the association frequencies summed over the first (R1), second (R2) and third (R3) response. Only responses that were also presented as cues are encoded in the network and all single loops were removed from this network. The resulting network corresponds to the largest weakly connected component, which corresponds maximal subset of nodes in a directed network that had at least one incoming or outgoing link^39^. Second, a sub-network (*G*_emotion_) that contained emotion word cues (*N* = 79) and their neighbors was extracted from the original network *G*_R123_. Multiple graph measures were calculated for the main graph and the emotion subgraph, including the number of nodes and edges, density, average shortest path, diameter, average cluster coefficients, average indegree, and outdegree. Third, to comprehend ​​synonymic Chinese emotion words such as 驚訝 (ging1ngaa6) and 驚喜 (ging1hei2) (both translated into *surprise*), cue-focused ego networks were extracted and emotional characteristics of their neighbors were compared.

#### Hubs and communities

For the emotion subgraph, hubs and communities were examined. Network hubs were defined as a node with the highest indegree and PageRank. Given a graph G, the PageRank algorithm computes a ranking of node *i* based on the structure of the incoming links:

$$PR_{i} = d\sum\limits_{{j \in B_{i} }} {\frac{{PR_{j} }}{{L_{j} }}} + \frac{1 - d}{N}$$

Communities were detected by the “*rb_pot*” algorithm implemented in the “*cdlib*” package. This algorithm is an extension of the established modularity maximization method with explicit use of the information contained in edge directions^40^. The mathematical representation states the following:

$$Q = \sum\limits_{ij} {\left( {Aij - \gamma \frac{{k_{i}^{out} k_{i}^{in} }}{m}} \right)} \delta (\sigma_{i} ,\sigma_{j} )$$

#### Robustness of communities

We constructed a null distribution of modularity score (Q-value) of randomly resampled graphs based on the original emotion graph to examine the robustness of community detection. This robustness test is also adopted by studies in other fields such as brain functional connectivity^41^. The Q-value measures the strength of community structure, which is the division of a graph into communities^42^. We randomly shuffled the edge weights of the original emotion graph to create 10,000 permutation graphs. The same community detection algorithm (“*rb_pot*”) was applied to each permutation graph and modularity scores were calculated. The robustness of the community detection was assessed by counting the number of larger Q-values of permutation graphs compared to the original emotion graph. *p*_*permutation*_ is determined by the count dividing by the total number of permutations (n = 10,000). If *p*_*permutation*_ < 0.05, it suggests that the communities of the emotion graph are statistically robust.

## Results

**Figure 1:**
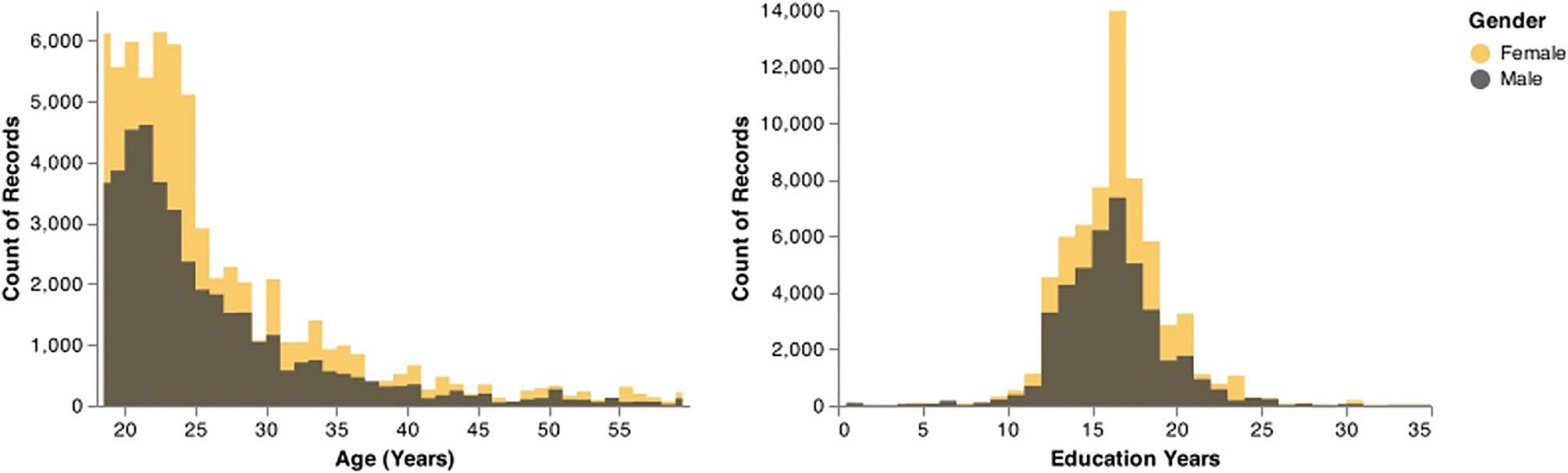
Distribution of age and education years by gender. Similar distribution of age and education years between males and females.

During the period 2014–2019, 10,693 participants completed the word association task through our website. After data preprocessing on cue words, 10,551 participants remained. 6497 healthy participants (60.4% are female, n = 3921) met our inclusion criteria in this cross-sectional semantic WAT task. The mean age was 25.5 ± 7.88 (male: 25.2 ± 7.56; female: 25.7 ± 8.08) with the average number of years of education being 15.8 ± 3.25 (male: 15.7 ± 3.24; female: 15.9 ± 3.26). Figure 1 demonstrates that female and male participants were distributed similarly in terms of age and years of education.

### Properties of emotional words

**Figure 2:**
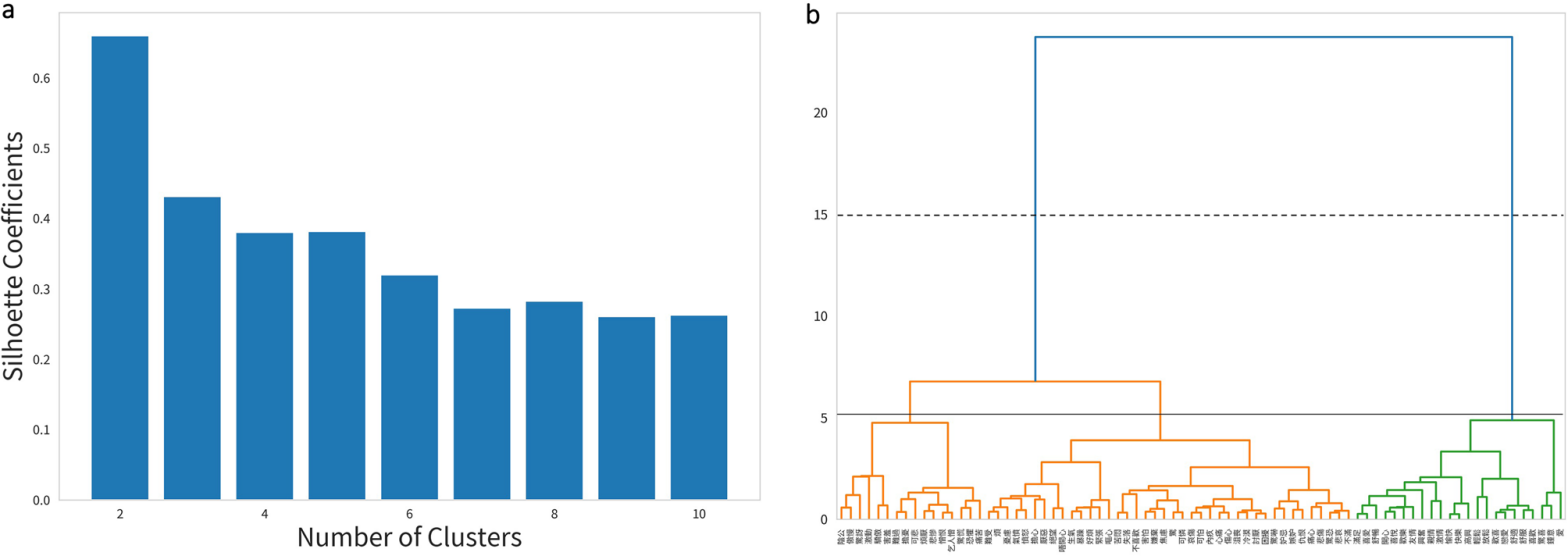
Hierarchical clustering based on the original ratings of valence, arousal, dominance and concreteness using Euclidean distance and Ward linkage function. (**a**) Silhouette coefficients by number of clusters. Two-cluster is the optimal number of clusters. (**b**) Dendrogram with two-cluster and three-cluster cut points, dash line is two-cluster and solid line is three-cluster.

**Figure 3:**
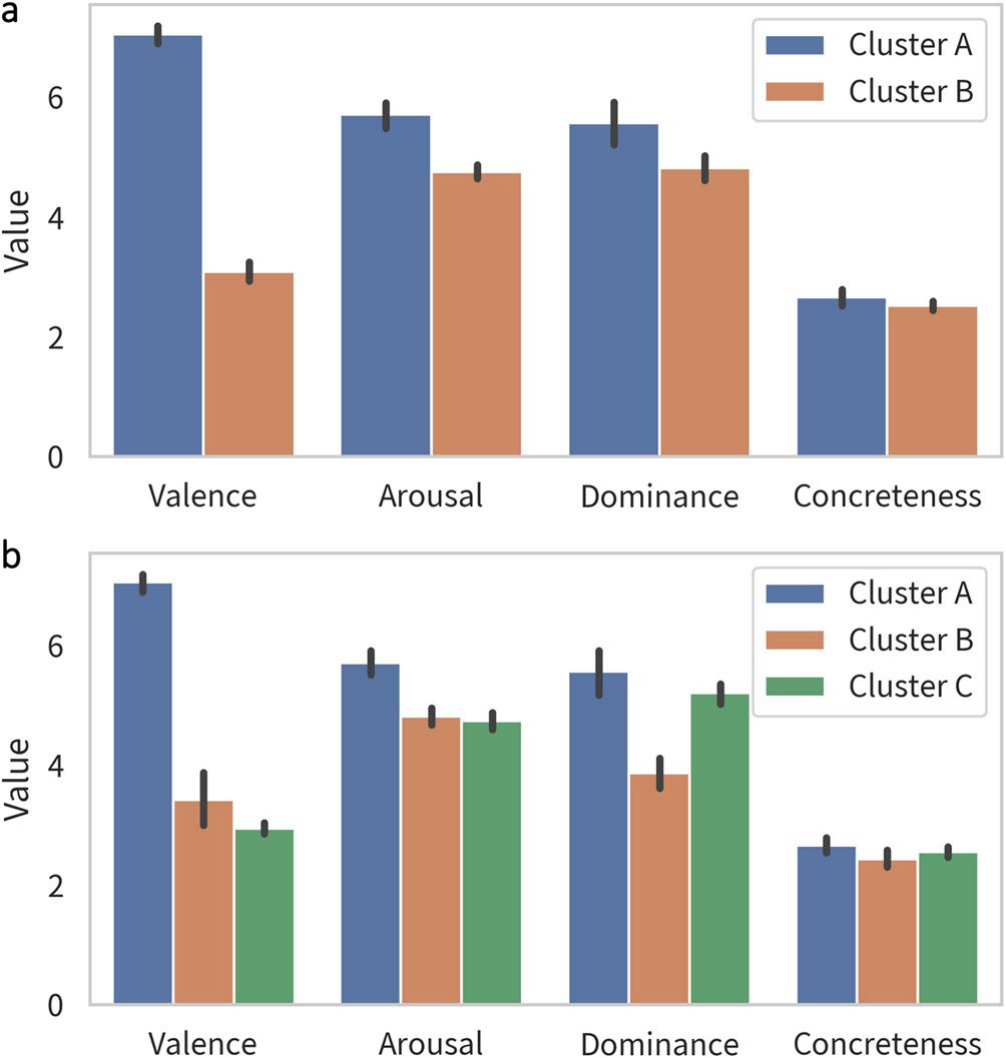
Descriptive profiles of each group in two-cluster and three-cluster solutions. (**a**) Group profile of two-cluster solution. Cluster A is positive and Cluster B is negative. (**b**) Group profile of three-cluster solution. Cluster A is positive, Cluster B is influenced negative, and Cluster C is influential negative.

On average, emotion cues elicited 16.5 number of responses per word. Hierarchical clustering with silhouette coefficients suggested that the optimal number of clusters is two. Most combinations of distance and linkage functions for hierarchical clustering obtained a similar result (see Figure S1). The clusters basically represented positive and negative emotions. For this study, we displayed both the 2- and 3-clustering solutions using the Euclidean distance and ward linkage function (Fig. 2). Hierarchical clustering with standardized scores was consistent compared to the ones with the original scores (see Figure S2 in supplementary materials). Figure 3 visualized the features of each cluster in two-cluster and three-cluster solutions. Summary descriptive of cluster valence, arousal, dominance, and concreteness was calculated by taking the geometric mean of subordinate emotion words (Table S3). In a 2-cluster solution, ratings on valence and arousal were higher in the positive emotion cluster (cluster A) than in the negative (cluster B). In the 3-cluster solution, ratings on dominance were higher in positive (cluster A) and influenced negative (cluster B) than in the influential negative (cluster C).

### Hubs and community detected in network analysis

The main graph (*G*_R123_) contained 2352 nodes with 75,141 edges while the emotion subgraph (*G*_emotion_) contained 864 nodes with 20,363 edges. The network statistics of each network are summarized in Table S4 (see supplementary materials). The *G*_emotion_ had a higher density than the *G*_R123_, which suggested that *G*_emotion_ is more heterogeneous in terms of connected nodes. This may reflect that emotions are associated with a different category (i.e., a syntagmatic response, e.g., fear-ghost instead of fear-afraid). Both *G*_R123_ and *G*_emotion_ demonstrated a small-world organization.

**Figure 4:**
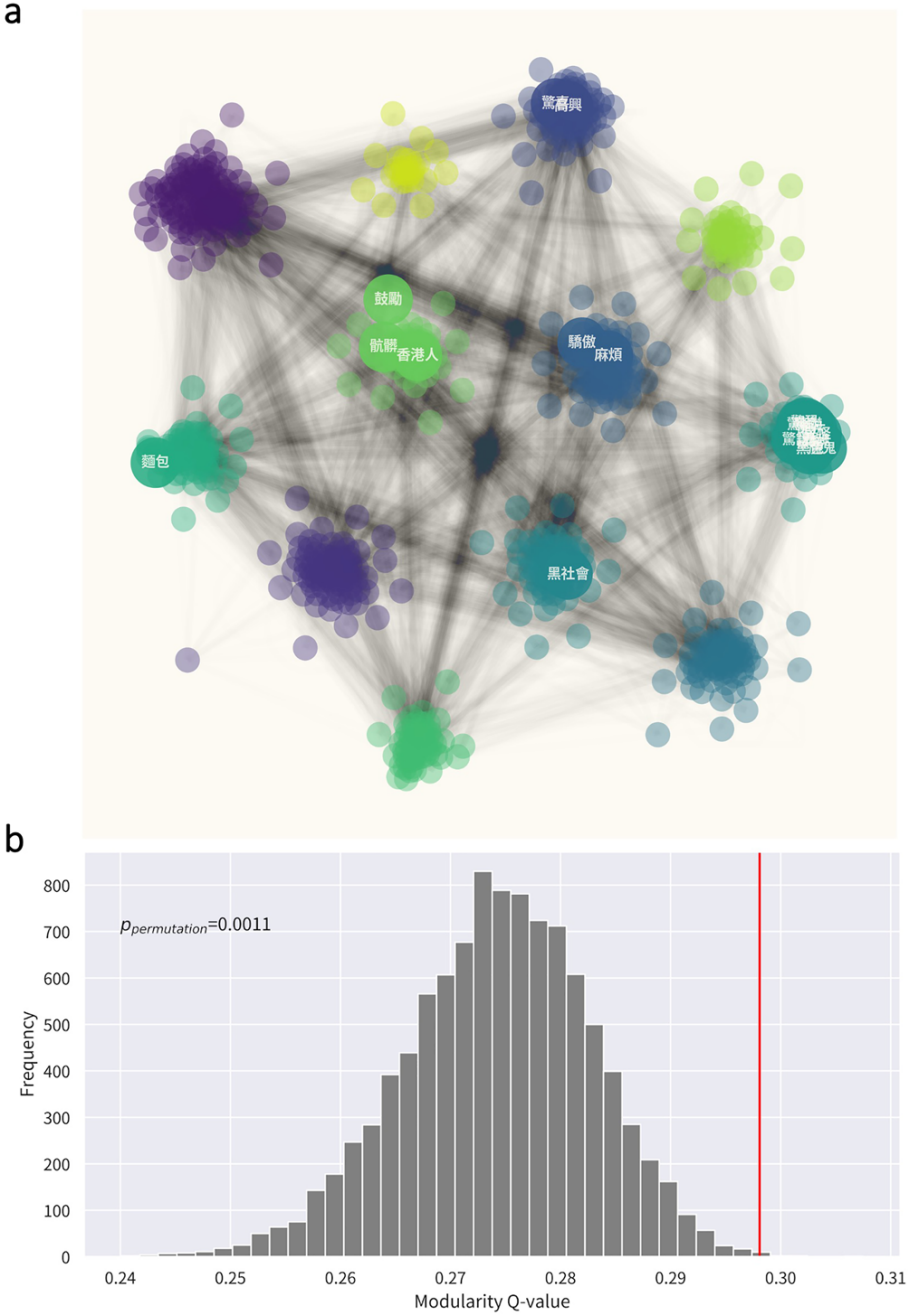
Hubs and community detected in network analysis and stability test. (**a**) Large-scale visualization of hubs and communities in the *G*emotion network. (**b**) Robustness of community detection. The histogram bars are modularity scores (Q-value) of 10,000 permutation graphs. The vertical line is the Q-value of the original emotion graph. *p*_permutation_ = 0.0011 suggested that the community structure of the emotion graph was robust.

Within the 10,000 iterations, 12 communities were the most frequent solutions. Figure 4a showed the macroscopic structure with the top 20 most central nodes. Using central nodes in terms of in-strength and PageRank with ⍺ set 0.8^43^, the nodes included 鬼 (*ghosts*), 鬼怪 (*ghosts and monsters*), 鬼魂 (*soul*), 黑暗 (*darkness*), 鬼片 (*ghost movies*), 黑色 (*black*) and 魔鬼 (*devil*) seemed to reflect fear of unknown and danger rooted from an evolutionary origin. These hubs were linked to 驚慌 (*thrilled,* ging1fong1), 驚恐 (*panic,* ging1hung2), and 驚訝 (*surprise,* ging1ngaa6) and formed a community. Table 2 summarized the 12 communities in the *G*_emotion_. Most communities could identify emotions with their relevant concepts under the same prototype. These emotion communities covered basic emotions such as love, happiness, sadness, anger, and fear.

**Figure 5:**
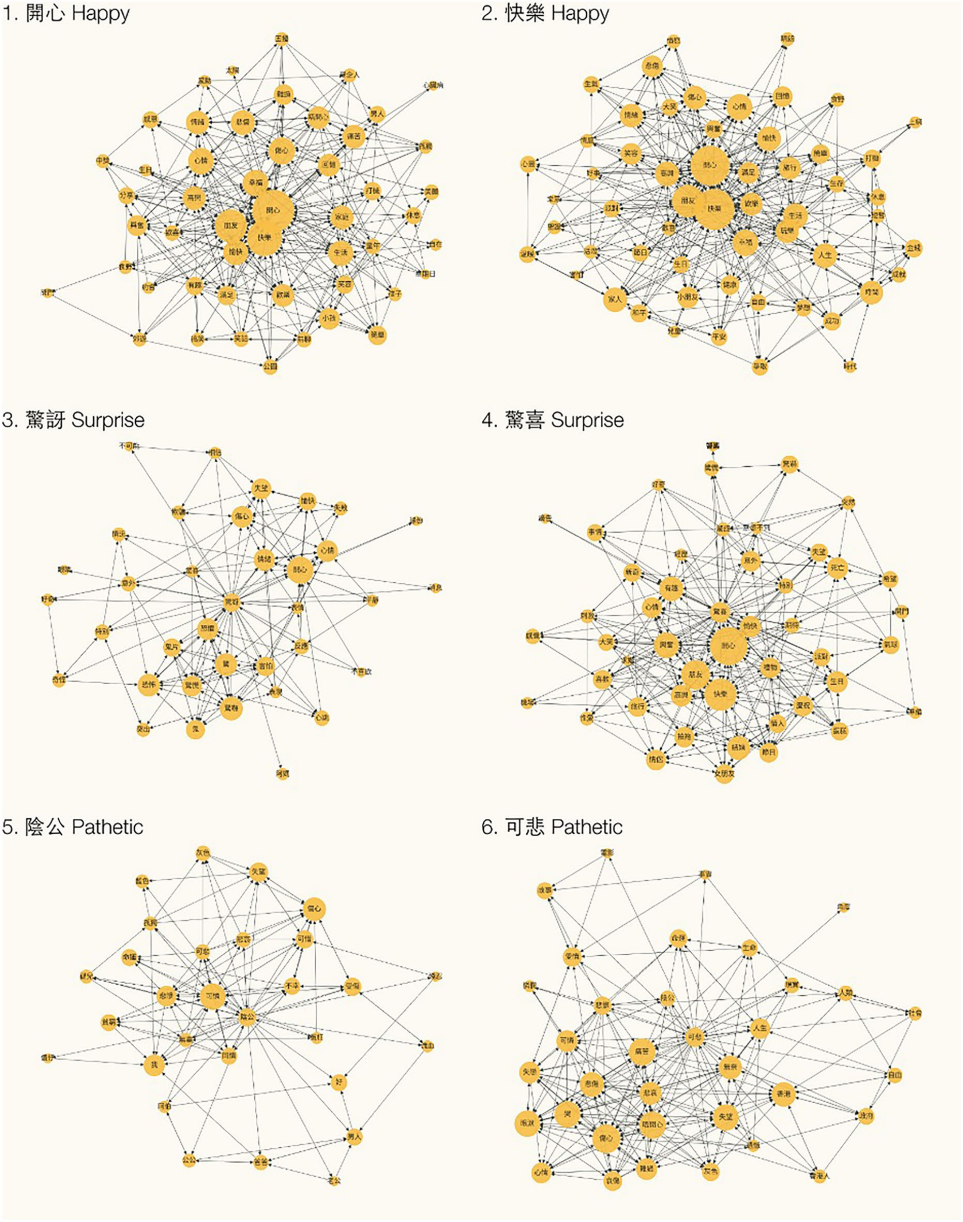
Cue-focused graph of similar emotion words.

**Table 2:** Communities in the *G*_emotion_.

Community	Label	Emotion-Label Words	Non Emotion-Label Words (Top 10 in-degree)*
1	Disturbance and worry	煩, 苦悶, 緊張, 困擾, 好煩	工作, 麻煩, 壓力, 考試, 辛苦, 失敗, 成功, 問題, 努力, 老師
2	Love	鍾意, 愛, 激情, 戀愛, 親情, 友情, 喜歡	朋友, 家人, 家庭, 父母, 媽媽, 感情, 關係, 女朋友, 結婚, 情人
3	Anger and hatred	仇恨, 冷漠, 煩厭, 厭惡, 乞人憎, 激動, 不喜歡, 討厭, 氣憤, 暴躁, 憎恨, 生氣, 憤怒	情緒, 警察, 思想, 受傷, 負面, 性格, 態度, 行為, 壞人, 殺人
4	Happiness	高興, 快樂, 愉快, 歡樂, 開心, 驚喜, 歡喜, 滿足, 喜愛, 興奮	心情, 紅色, 遊戲, 感覺, 有趣 音樂, 笑容, 電視, 玩樂, 打機
5	Pride	羞, 驕傲, 嫉妒, 妒忌, 傲慢	女人, 男人, 我, 自己, 小朋友 小孩, 可愛, 你, 女性, 美麗
6	Sadness	悲哀, 唔開心, 內疚, 可悲, 失落, 悲傷, 傷心, 痛心, 絕望, 難受, 心痛, 難過, 嫌棄, 悲慘, 可憐, 沮喪, 痛苦, 哀傷, 陰功	哭泣, 哭, 無奈, 分手, 自殺 眼淚, 失去, 孤獨, 失戀, 離開
7	Relaxedness	舒適, 輕鬆, 放鬆, 舒暢, 舒服	生活, 自由, 旅行, 休息, 享受, 睡覺, 環境, 日本, 放假, 太陽
8	Fear and disgust	驚, 恐懼, 嘔心, 憂慮, 焦慮, 害怕, 驚慌, 驚嚇, 驚恐, 驚訝, 可怕, 擔憂, 擔心	死亡, 電影, 黑暗, 危險, 安全, 事件, 黑色, 意外, 神, 鬼
9	Discontent	不滿	香港, 政府, 政治, 社會, 世界, 垃圾, 和平, 地方
10	Wellbeing		健康, 精神, 醫生, 醫院, 運動, 身體, 病人, 精神病, 疾病, 藥物
11	Essentials		時間, 金錢, 生命, 食物, 重要, 人物, 珍惜, 付出, 故事, 美食
12	Life		人生, 回憶, 未來, 夢想, 希望, 美好, 理想, 過去, 現實, 將來

*Please refer to Table S5 in supplementary material for the Cantonese pinyin and translation.

The robustness test showed that only a few permutation graphs can achieve the same or higher modularity Q-value compared to the original graph (Fig. 4b, *p*_permutation_ = 0.0011). This suggested that the community structure of the emotion graph is robust and unlikely to be a random result.

### Microscopic properties: degree of emotion expression in association graph

Even similar emotion cue words represent different degrees of emotion. Will the related nodes of similar emotion words have different emotional properties ratings? To test this hypothesis, we first extracted ego networks for each emotion word. Next, we calculated the mean emotional characteristics of unique neighbors of each graph. Two-sample t-tests rejected the null hypothesis that there is any difference between 開心 (hoi1sam1, Fig. 5.1) and 快樂 (faai3lok6, Fig. 5.2) (both translated into *happy*, p > 0.05) or 陰功 (jam1gung1, Fig. 5.5) and 可悲 (ho2bei1, Fig. 5.6) (both translated into *pathetic*, p > 0.05). We further tested if they are statistically equivalent using Equivalence Test (i.e., two one-sided test, TOST) with a boundary between − 0.5 and 0.5^44^. Results showed that 開心 (hoi1sam1) and 快樂 (faai3lok6) were statistically equivalent in valence (lower bound: t = 0.68, p = 0.249; upper bound: t = − 2.87, p = 0.002), arousal (lower bound: t = 4.06, p < 0.001; upper bound: t = − 4.26, p < 0.001), dominance (lower bound: t = 2.42, p = 0.009; upper bound: t = − 4.93, p < 0.001) and concreteness (lower bound: t = 4.27, p < 0.001; upper bound: t = − 2.69, p = 0.004). 陰功 (jam1gung1) and 可悲 (ho2bei1) were statistically equivalent in arousal (lower bound: t = 2.39, p = 0.01; upper bound: t = − 4.40, p < 0.001), dominance (lower bound: t = 1.19, p = 0.12; upper bound: t = − 2.95, p = 0.002), concreteness (lower bound: t = 3.39, p < 0.001; upper bound: t = − 0.56, p = 0.28) but not valence (lower bound: t = 0.74, p = 0.23; upper bound: t = − 1.55, p = 0.06). In Cantonese, 驚訝 (ging1ngaa6) and 驚喜 (ging1hei2) can be translated into surprise. In our hierarchical clustering, these two cues were assigned to two different clusters. 驚喜 (*surprise,* ging1hei2) is clustered as positive while 驚訝 (*surprise,* ging1ngaa6) is clustered as influenced negative (Table S2). Particularly, we observed that the hub (measured by in-degree) in the 驚喜 (*surprise,* ging1hei2) graph (Fig. 5.4) is 開心 (*happiness*, hoi1sam1). This is further supported by the average rating of unique nodes in these two graphs. The nodes in the 驚喜 graph (Fig. 5.4) showed to have higher valence (Δ mean = 1.65, t = 5.28, p < 0.001, Cohen's d = 1.36) and higher arousal (Δ mean = ​​0.61, t = 4.02, p < 0.001, Cohen's d = 1.03) as well as more concrete (Δ mean = ​​0.59, t = 3.16, p = 0.002, Cohen's d = 0.81) compared to those in the 驚訝 graph (Fig. 5.3). This implied that though both words translated into *surprise* in English, 驚喜 (*surprise*, ging1hei2) invokes more positive, exciting, and concrete feelings compared to 驚訝 (*surprise,* ging1ngaa6).

## Discussion

The current study explored the structure and organization of the mental lexicon of Cantonese emotion words and their related concepts. First, we described the dimensions of emotion word cues using hierarchical clustering. Further, we explored the network properties at different levels of an emotion network *G*_emotion_ constructed from a free association task. Our results showed that *G*_emotion_ demonstrated that emotions and relevant concepts were grouped in a single cluster. Relying only on lexicons and clustering procedures in the current study, the network approach resulted in more distinct clusters of emotion lexicon compared to hierarchical clustering. The linkage between emotion labels and relevant conceptual knowledge could be evolutionarily rooted (e.g., fear and darkness) or recently formed (e.g., discontent and policies). We also identified three communities without emotion labels. These communities may represent emotion-laden words that are closely connected to emotion labels. Finally, we examined the microscopic properties of certain emotion labels. The results revealed that the abilities of emotion graphs with concepts can differentiate emotion labels with similar meanings, such as nodes connected to synonyms of surprise (驚喜ging1hei2, 驚訝ging1ngaa6) were different from each other.

Languages that dominate western cultures (e.g., English) are mostly spoken in low-context cultures, while the languages that dominate eastern cultures (e.g., Mandarin, Cantonese) are generally used in high-context cultures. Words in Western cultures generally have universal meanings and people pay less attention to the communication context^45^. People with Eastern cultural backgrounds are more likely to communicate beyond words and use the same word differently in specific contexts^45^. In hierarchical clustering, the contextual profiles related to each emotion category are missing. For example, words from the same emotion types were assigned to different clusters, such as 煩厭 (*boredom*, faan4jim3) in cluster B, while 厭惡 (*disgusted*, jim3wu3), 討厭 (*dislike*, tou2 jim3) in cluster C. Besides, words with the same English translation were identified in different clusters. This partial semantic equivalence between English and Cantonese was demonstrated specifically for *surprise* (驚喜ging1hei2, 驚訝ging1ngaa6). The fuzziness in the boundaries of emotion clusters in hierarchical clustering analysis may be due to the communication custom in Chinese. For the same emotion cue words, the responders may conceptualize them in a specific context that varies among people. If emotions were categorized in terms of affective features, we only identified two superordinate clusters covering a general positive–negative dimension. In the setting of our study, hierarchical clustering could not offer a precise clustering of emotion labels. This may be due to the unique characteristics of Chinese emotion-label words. However, further analysis in English using the same settings is needed to confirm these findings. Comparing clustering using ratings, an emotion graph can better differentiate emotions into relevant prototypical ones. Altogether, more work in which clustering solutions across languages are compared is needed to corroborate these claims.

At a macroscopic level, the *G*_emotion_ has a small-world structure which was characterized by short averaged shortest path-lengths and a significant degree of clustering (Table S4 in supplementary material). Along with previous reports, *G*_emotion_, as a mental graph, its hubs expressed psychological importance^15^. Some associations between emotions and non-emotion words are “inherited” while some of the others are linked recently. For example, 黑暗 (*darkness*) and 黑色 (*black*) were grouped with 驚 (fear). This is consistent with previous research that proposed fear is an inherent default response to the unknown and the fear response is disinhibited under uncertainties (e.g., dark environment)^46,47^. Besides, the association may reflect evolutionary importance for survival^48^. For example, 黑暗 (*darkness*) and 黑色 (*black*) indicate potential threats and dangers, and this aligns with previous research on the association between fear and the color black^49^. The conceptual knowledge of fear linking to threatening instances may have a long history tracing back to our ancestors. Recent instances could generate a strong linkage to a specific emotion, which forms the generated associations. For example, since 2014, Hong Kong has been going through a series of events causing social unrest. We found that the emotion-label word 不滿 (*discontent*) was grouped with cues including government, society, and politics. Excessive violent acts by the police during social unrest may lead to an association between 警察 (police) and emotion labels in anger and hatred. These examples were consistent with recent findings reporting social unrest in Hong Kong was associated with affective disturbances in residents^50,51^.

Three communities without emotion-label words were documented, namely well-being, essentials, and life. These communities did not involve a specific emotion label and were composite of only emotion-laden words (e.g., 醫院, hospital and 夢想, dreams). Compared to emotion-label words, emotion-laden words trigger emotions without explicitly elucidating an affective state. Previous evidence suggested that emotion-laden words had distinct brain mechanisms compared to emotion-label ones^52^. These words do not have a specific linkage to any emotion labels so they may be contextual dependent. For example, death and birth in relation to the hospital can represent two extremes of the valence dimension.

The microscopic structure of emotion words can offer insights into their relative concepts. As illustrated in our study, 驚喜 (*surprise*, ging1hei2) and 驚訝 (*surprise,* ging1ngaa6) have the same root word 驚 (*fear,* ging1) but the combination of 喜 (*happiness,* hei2) leads to a positive valence while that of 訝 (*surprise,* ngaa6) leads to a negative one. The add-on characters seem to drive the vocabularies into two ends in the dimension of valence. The emotion graphs further illustrate that the microstructure of these two words is different in conceptual knowledge. As in Fig. 5.4, 驚喜 (*surprise with happiness,* ging1hei2) is linked to 情人 (*lover*), 女朋友 (*girlfriend*), and 生日 (*birthday*) as well as emotion labels related to happiness (e.g., 開心 *happy*, hoi1sam1). Instead, the 驚訝 (*surprise with fear*, ging1ngaa6) graph clearly showed that it is linked to fear-related emotion (e.g., 恐懼 *fear*, hung2geoi6). We expected that 開心 (*happy*, hoi1sam1) and 快樂 (*happy,* faai3lok6) might differ in terms of concreteness but we found no difference. Instead, we observed overlapping patterns of graphs. This may suggest that in daily usage, these two phrases may be used to express almost identical meanings even though they are lexically different.

When studying words that are unique to a language and not strictly emotion labels, we can explore their relationships with prototypical emotions to understand the meaning they carry. In the current study, we used 陰功 (*pathetic,* jam1gung1) as an example of those words in Cantonese. 陰功 (*pathetic*, jam1gung1) expressed a feeling of sadness and pity. We found that there is no difference between 陰功 (*pathetic*, jam1gung1) and 可悲 (*pathetic*, ho2 bei1), suggesting our graphs are sensitive to examining the relationship between metaphoric emotion labels and prototypical emotions.

Although the current study illustrated the usefulness of studying emotions in a free word association graph. There are several limitations worth discussing. First, as this study only covered a subset of the SWOW-HK study, the emotion word list may not cover the full spectrum of Chinese emotion labels. A previous study identified 953 emotion words over 3766 words^22^ using Pavlenko’s framework^53^. Second, our participants were mostly people younger than 30 years of age who used Cantonese since the recruitment process was conducted through popular online platforms in Hong Kong where the main users are usually younger local people. It is possible that the mental lexicon network we found may not adequately represent the older population and those without access to the internet. Further, although our findings suggested a difference in cultural contexts between Cantonese and English, the current study does not include the analysis of English emotion lexicons given the limited data. Future cross-language comparisons such as the study conducted by Thornton and colleagues^54^ would be beneficial to evaluate this argument. Further, the current findings relied on the self-report data and clustering algorithms chosen in the study. More work could be done to validate the robustness of the current results by applying different algorithms to other types of data, such as online text data and emotional task scores^55,56^, to validate the robustness of current results.

In sum, our results demonstrate that mental lexicon graphs constructed from free associations of emotion-label words can reveal fine communities of emotions and their relevant concepts. These graphs are also able to differentiate the relevant emotion concepts which directly link to emotion labels from emotion-laden words that may be contextually dependent. Furthermore, emotion concepts can be formed distally and proximally as demonstrated by fear and discontent communities. Future emotion studies may utilize this approach to study the role of language in human emotion.

## Supplementary Information

Supplementary Information.

## Data Availability

The datasets generated and analyzed during the current study are available from the corresponding authors on reasonable request.

## Code availability

All statistical analyses and visualization were performed with Python version 3.8.8. Network analyses were implemented with packages including “networkx” and “cdlib” while visualization was implemented with packages including “netgraph” and “altair”. Code and details can be found on the Github (https://github.com/kamione/wat_emotion_graph).
