# Supplementary material for: Discovering the structure and organization of a free Cantonese emotion-label word association graph to understand mental lexicons of emotions
Source: Sci Rep. 2022 Nov 15;12:19581. doi: 10.1038/s41598-022-23995-z (PMC9666539; doi:10.1038/s41598-022-23995-z)
Supplement: Supplementary file 1 — Supplementary Information. [file 41598_2022_23995_MOESM1_ESM.docx]

**Discovering the structure and organization of a free Cantonese emotion-label word association to understand mental lexicons of emotions**

Ting Yat Wong^1,2,^*^,^**^+^**, Zhiqian Fang^1,^*, Yat To Yu^1^, Charlton Cheung^1^, Christy LM Hui^1^, Brita Elvevåg^3^, Simon De Deyne^4^, Pak Chung Sham^1,5^, Eric YH Chen^1,5,+^

1. Department of Psychiatry, School of Clinical Medicine, Li Ka Shing Faculty of Medicine, University of Hong Kong, Hong Kong
2. Neurodevelopment and Psychosis Section, Department of Psychiatry, Perelman School of Medicine, University of Pennsylvania, Philadelphia, USA
3. Department of Clinical Medicine, University of Tromsø – the Arctic University of Norway, Tromsø, Norway
4. School of Psychological Sciences, University of Melbourne
5. State Key Laboratory of Brain and Cognitive Sciences, University of Hong Kong, Hong Kong

* These authors contributed equally

**^+^ Corresponding Authors:** Ting Yat Wong ([tywong.one@gmail.com](mailto:tywong.one@gmail.com)); Eric YH Chen ([eyhchen@hku.hk](mailto:eyhchen@hku.hk))

**Supplementary material**

**Table S1. Emotion properties of 79 Cantonese emotion words**

| **Chinese** | **Cantonese Pinyin** | **Translation** | **Valence** | **Arousal** | **Dominance** | **Concreteness** |
| --- | --- | --- | --- | --- | --- | --- |
| 友情 | jau5 cing5 | friendship | 6.80 | 5.90 | 6.44 | 2.65 |
| 喜悅 | hei2 jyut6 | joy | 7.35 | 5.80 | 6.38 | 2.35 |
| 喜愛 | hei2 oi3 | favourite | 7.00 | 5.50 | 6.13 | 2.35 |
| 喜歡 | hei2 fun1 | like | 7.20 | 5.45 | 5.06 | 3.20 |
| 快樂 | faai3 lok6 | happiness | 7.60 | 6.25 | 6.06 | 3.05 |
| 愉快 | jyu4 faai3 | happy | 7.55 | 6.30 | 5.94 | 3.25 |
| 愛 | oi3 | love | 7.20 | 6.50 | 3.88 | 2.20 |
| 戀愛 | lyun2 oi3 | love | 6.95 | 5.35 | 5.38 | 2.90 |
| 放鬆 | fong3 sung1 | relaxed | 6.50 | 4.25 | 5.63 | 2.35 |
| 歡喜 | fun1 hei2 | joy | 7.20 | 5.50 | 5.38 | 2.75 |
| 歡樂 | fun1 lok6 | joy | 7.25 | 5.70 | 6.38 | 2.95 |
| 滿足 | mun5 zuk1 | satisfied | 7.15 | 5.70 | 6.06 | 2.30 |
| 激情 | gik1 cing4 | passion | 5.95 | 6.05 | 5.75 | 2.45 |
| 興奮 | hing1 fan5 | excited | 6.75 | 6.20 | 7.13 | 2.50 |
| 舒暢 | syu1 coeng3 | comfortable | 6.85 | 5.10 | 6.00 | 2.50 |
| 舒服 | syu1 fuk6 | comfortable | 7.20 | 5.75 | 5.19 | 2.90 |
| 舒適 | syu1 sik1 | comfortable | 6.95 | 5.50 | 5.13 | 2.55 |
| 親情 | can1 cing4 | family affection | 6.80 | 6.10 | 5.63 | 2.65 |
| 輕鬆 | hing1 sung1 | relaxed | 7.05 | 4.95 | 5.06 | 2.05 |
| 鍾意 | zung1 ji3 | like | 7.00 | 6.00 | 3.56 | 2.85 |
| 開心 | hoi1 sam1 | happiness | 7.25 | 6.00 | 6.38 | 2.65 |
| 驚喜 | ging1 hei2 | surprise | 6.90 | 5.40 | 3.75 | 3.00 |
| 高興 | gou1 hing1 | happy | 7.70 | 5.95 | 5.75 | 2.65 |
| 乞人憎 | hat1 jan4 zang1 | loathsome | 2.65 | 4.85 | 3.94 | 2.50 |
| 傲慢 | ngou6 maan6 | arrogant | 3.40 | 5.00 | 4.06 | 2.80 |
| 可悲 | ho2 bei1 | pathetic | 3.20 | 4.50 | 3.40 | 1.90 |
| 害羞 | hoi6 sau1 | shy | 5.15 | 4.90 | 3.81 | 2.50 |
| 失落 | sat1 lok6 | down | 3.35 | 4.05 | 4.56 | 2.45 |
| 恐懼 | hung2 geoi6 | fear | 2.65 | 4.70 | 3.38 | 2.75 |
| 悲傷 | bei1 soeng1 | sadness | 2.65 | 4.45 | 4.69 | 3.10 |
| 悲哀 | bei1 oi1 | sorrow | 3.20 | 4.80 | 4.32 | 2.90 |
| 悲慘 | bei1 caam2 | miserable | 2.70 | 5.25 | 3.88 | 2.30 |
| 憎恨 | zang1 han6 | hatred | 2.85 | 4.85 | 3.88 | 2.25 |
| 擔憂 | daam1 jau1 | worry | 2.95 | 4.40 | 4.13 | 2.20 |
| 激動 | gik1 dung6 | excited | 5.05 | 4.95 | 5.25 | 3.10 |
| 煩厭 | faan4 jim3 | boredom | 3.05 | 5.40 | 4.00 | 2.35 |
| 痛心 | tung3 sam1 | agonised | 2.70 | 4.95 | 4.44 | 2.90 |
| 痛苦 | tung3 fu2 | agony | 2.30 | 4.80 | 3.25 | 2.00 |
| 苦悶 | fu2 mun6 | bored | 3.45 | 4.15 | 4.81 | 2.30 |
| 陰功 | jam1 gung1 | miserable | 3.75 | 4.75 | 4.19 | 2.45 |
| 難過 | naan4 gwo3 | upset | 2.70 | 4.35 | 4.13 | 2.45 |
| 驕傲 | giu1 ngou6 | proud | 4.90 | 4.85 | 3.19 | 2.40 |
| 驚慌 | ging1 fong1 | frightened | 2.89 | 5.00 | 3.06 | 2.50 |
| 驚訝 | ging1 ngaa6 | surprised | 4.45 | 4.40 | 4.25 | 2.45 |
| 不喜歡 | bat1 hei2 fun1 | dislike | 3.20 | 3.95 | 5.25 | 3.25 |
| 不滿 | bat1 mun5 | dissatisfied | 3.00 | 4.6o | 4.56 | 2.85 |
| 仇恨 | sau4 han6 | hatred | 2.50 | 4.70 | 4.81 | 2.40 |
| 傷心 | soeng1 sam1 | sad | 2.65 | 4.60 | 5.31 | 2.70 |
| 冷漠 | laang5 mok6 | indifferent | 2.80 | 4.05 | 4.88 | 2.40 |
| 內疚 | noi6 gau3 | guilty | 2.95 | 4.15 | 5.31 | 2.55 |
| 厭惡 | jim3 wu3 | disgusted | 2.75 | 4.00 | 5.94 | 2.60 |
| 可怕 | ho2 paa3 | terrified | 2.95 | 4.65 | 5.25 | 2.20 |
| 可憐 | ho2 lin4 | miserable | 3.55 | 4.70 | 5.50 | 2.30 |
| 哀傷 | oi1 soeng1 | sad | 2.80 | 4.45 | 5.38 | 2.35 |
| 唔開心 | m4 hoi1 sam1 | sad | 2.35 | 5.00 | 5.50 | 2.50 |
| 嘔心 | au2 sam1 | disgusted | 3.15 | 5.40 | 4.88 | 3.10 |
| 困擾 | kwan3 jiu5 | distressed | 3.05 | 4.30 | 4.94 | 2.35 |
| 好煩 | hou2 faan4 | very annoying | 3.10 | 5.25 | 5.38 | 2.40 |
| 妒忌 | dou3 gei6 | jealous | 2.90 | 5.05 | 4.69 | 2.25 |
| 嫉妒 | zat6 dou3 | jealousy | 2.65 | 5.05 | 5.06 | 2.50 |
| 嫌棄 | jim4 hei3 | despise | 3.05 | 4.55 | 5.00 | 2.60 |
| 害怕 | hoi6 paa3 | scared | 3.40 | 4.45 | 5.06 | 2.55 |
| 心痛 | sam1 tung3 | heartbroken | 2.50 | 4.50 | 5.25 | 2.45 |
| 憂慮 | jau1 leoi6 | worry | 2.89 | 4.55 | 5.94 | 1.94 |
| 憤怒 | fan5 nou6 | angry | 2.85 | 5.00 | 5.94 | 2.95 |
| 擔心 | daam1 sam1 | worry | 3.05 | 4.60 | 6.63 | 2.55 |
| 暴躁 | bou6 cou3 | irritable | 3.25 | 5.65 | 5.50 | 2.65 |
| 氣憤 | hei3 fan5 | angry | 3.00 | 5.05 | 5.81 | 2.55 |
| 沮喪 | zeoi2 song3 | frustrated | 2.55 | 4.25 | 4.94 | 2.35 |
| 焦慮 | ziu1 leoi6 | anxious | 3.10 | 4.60 | 5.38 | 2.80 |
| 煩 | faan4 | annoyed | 2.85 | 4.85 | 5.94 | 2.20 |
| 生氣 | sang1 hei3 | angry | 3.00 | 5.60 | 5.25 | 2.80 |
| 絕望 | zyut6 mong6 | despair | 2.15 | 5.40 | 5.44 | 2.20 |
| 緊張 | gan2 zoeng1 | nervous | 3.40 | 5.75 | 5.25 | 2.35 |
| 討厭 | tou2 jim3 | dislike | 2.80 | 4.35 | 4.88 | 2.40 |
| 難受 | naan4 sau6 | unhappy | 2.60 | 4.85 | 6.13 | 2.40 |
| 驚 | geng1 | shock | 3.40 | 4.95 | 5.44 | 2.45 |
| 驚嚇 | ging1 haak3 | startled | 3.05 | 5.35 | 4.50 | 2.60 |
| 驚恐 | ging1 hung2 | panicked | 3.10 | 4.60 | 4.19 | 2.70 |

**Table S2. Emotion word cues in 3 data-driven clusters**

|  | **Cluster A** | **Cluster B** | **Cluster C** |
| --- | --- | --- | --- |
| **Two-Cluster**  **Emotion Words** | 友情, 喜悅, 喜愛, 喜歡, 快樂, 愉快, 愛, 戀愛, 放鬆, 歡喜, 歡樂, 滿足, 激情, 興奮, 舒暢, 舒服, 舒適, 親情, 輕鬆, 鍾意, 開心, 驚喜, 高興 | 乞人憎, 傲慢, 可悲, 害羞, 恐懼, 悲慘, 憎恨, 擔憂, 激動, 煩厭, 痛苦, 陰功, 難過, 驕傲, 驚慌, 驚訝, 不喜歡, 不滿, 仇恨, 傷心, 內疚, 冷漠, 厭惡, 可怕, 可憐, 哀傷, 唔開心, 嘔心, 困擾, 失落, 好煩, 妒忌, 嫉妒, 嫌棄, 害怕, 心痛, 悲傷, 悲哀, 憂慮, 憤怒, 擔心, 暴躁, 氣憤, 沮喪, 焦慮, 煩, 生氣, 痛心, 絕望, 緊張, 苦悶, 討厭, 難受, 驚, 驚嚇, 驚恐 |  |
| **Three-Cluster**  **Emotion Words** | 友情, 喜悅, 喜愛, 喜歡, 快樂, 愉快, 愛, 戀愛, 放鬆, 歡喜, 歡樂, 滿足, 激情, 興奮, 舒暢, 舒服, 舒適, 親情, 輕鬆, 鍾意, 開心, 驚喜, 高興 | 乞人憎, 傲慢, 可悲, 害羞, 恐懼, 悲慘, 憎恨, 擔憂, 激動, 煩厭, 痛苦, 陰功, 難過, 驕傲, 驚慌, 驚訝 | 不喜歡, 不滿, 仇恨, 傷心, 內疚, 冷漠, 厭惡, 可怕, 可憐, 哀傷, 唔開心, 嘔心, 困擾, 失落, 好煩, 妒忌, 嫉妒, 嫌棄, 害怕, 心痛, 悲傷, 悲哀, 憂慮, 憤怒, 擔心, 暴躁, 氣憤, 沮喪, 焦慮, 煩, 生氣, 痛心, 絕望, 緊張, 苦悶, 討厭, 難受, 驚, 驚嚇, 驚恐 |

| Two clusters | **Cluster A** | **Cluster B** |  |
| --- | --- | --- | --- |
| **Cluster Label** | Positive | Negative |  |
| **Valence** | 7.05 (0.37) | 3.08 (0.60) |  |
| **Arousal** | 5.70 (0.50) | 4.75 (0.42) |  |
| **Dominance** | 5.57 (0.89) | 4.82 (0.80) |  |
| **Concreteness** | 2.65 (0.32) | 2.51 (0.28) |  |
| Three clusters | **Cluster A** | **Cluster B** | **Cluster C** |
| **Cluster Label** | Positive | Influenced Negative | Influential Negative |
| **Valence** | 7.05 (0.37) | 3.41 (0.95) | 2.94 (0.32) |
| **Arousal** | 5.70 (0.50) | 4.80 (0.30) | 4.73 (0.47) |
| **Dominance** | 5.57 (0.89) | 3.87 (0.53) | 5.20 (0.53) |
| **Concreteness** | 2.65 (0.32) | 2.43 (0.29) | 2.55 (0.28) |

**Table S3. Details of two and three data-driven clusters of emotion word cues**

*Please refer to Table S2 in supplementary material for the emotion word cues in each cluster.

**Table S4. Descriptive network statistics for the *G*_main_ and *G*_emotion_ networks**

|  | ***G*_main_** | ***G*_emotion_** |
| --- | --- | --- |
| Node N | 2,352 | 859 |
| Edge N | 75,141 | 20,386 |
| *Density* | 0.014 | 0.027 |
| *𝐿* | 2.81 | 2.64 |
| *max(L)* | 6 | 5 |
| *CC* | 0.0057 | 0.0075 |
| *k^in^* | 31.9 (35.9) | 23.7 (23.1) |
| *k^out^* | 31.9 (10.5) | 23.7 (9.63) |

**Table S5. Cantonese pinyin and translation of non-emotion-label words**

| **Chinese** | **Cantonese Pinyin** | **Translation** |
| --- | --- | --- |
| 工作 | gung1 zok3 | work |
| 麻煩 | maa4 faan4 | trouble |
| 壓力 | aat3 lik6 | stress |
| 考試 | haau2 si3 | test |
| 辛苦 | san1 fu2 | hard |
| 失敗 | sat1 baai6 | failure |
| 成功 | sing4 gung1 | success |
| 問題 | man6 tai4 | question |
| 努力 | nou5 lik6 | hard-working |
| 老師 | lou5 si1 | teacher |
| 朋友 | pang4 jau5 | friend |
| 家人 | gaa1 jan4 | family member |
| 家庭 | gaa1 ting4 | family |
| 父母 | fu6 mou5 | parents |
| 媽媽 | maa1 maa1 | mother |
| 感情 | gam2 cing4 | sentience |
| 關係 | gwaan1 hai6 | relationship |
| 女朋友 | neoi5 pang4 jau5 | girlfriend |
| 結婚 | git3 fan1 | marry |
| 情人 | cing4 jan4 | lover |
| 情緒 | cing4 seoi5 | emotion |
| 警察 | ging2 caat3 | police |
| 思想 | si1 soeng2 | thought |
| 受傷 | sau6 soeng1 | hurt |
| 負面 | fu6 min6 | negative |
| 性格 | sing3 gaak3 | personality |
| 態度 | taai3 dou6 | attitude |
| 行為 | hang4 wai4 | behaviour |
| 壞人 | waai6 jan4 | villian |
| 殺人 | saat3 jan4 | murder |
| 心情 | sam1 cing4 | mood |
| 紅色 | hung4 sik1 | red |
| 遊戲 | jau4 hei3 | game |
| 感覺 | gam2 gok3 | feeling |
| 有趣 | jau5 ceoi3 | interesting |
| 音樂 | jam1 ngok6 | music |
| 笑容 | siu3 jung4 | smile |
| 電視 | din6 si6 | television |
| 玩樂 | wun6 lok6 | play |
| 打機 | daa2 gei1 | play video games |
| 女人 | neoi5 jan4 | woman |
| 男人 | naam4 jan4 | man |
| 我 | ngo5 | I |
| 自己 | zi6 gei2 | myself |
| 小朋友 | siu2 pang4 jau5 | child |
| 小孩 | siu2 haai4 | child |
| 可愛 | ho2 oi3 | cute |
| 你 | nei5 | you |
| 女性 | neoi5 sing3 | female |
| 美麗 | mei5 lai6 | beautiful |
| 哭泣 | huk1 jap1 | weep |
| 哭 | huk1 | cry |
| 無奈 | mou4 noi6 | resignedly |
| 分手 | fan1 sau2 | break up |
| 自殺 | zi6 saat3 | suicide |
| 眼淚 | ngaan5 leoi6 | tears |
| 失去 | sat1 heoi3 | lose |
| 孤獨 | gu1 duk6 | lonely |
| 失戀 | sat1 lyun2 | breakup |
| 離開 | lei4 hoi1 | leave |
| 生活 | sang1 wut6 | life |
| 自由 | zi6 jau4 | freedom |
| 旅行 | leoi5 hang4 | travel |
| 休息 | jau1 sik1 | rest |
| 享受 | hoeng2 sau6 | enjoy |
| 睡覺 | seoi6 gaau3 | sleep |
| 環境 | waan4 ging2 | environment |
| 日本 | jat6 bun2 | Japan |
| 放假 | fong3 gaa3 | vacation |
| 太陽 | taai3 joeng4 | Sun |
| 死亡 | sei2 mong4 | death |
| 電影 | din6 jing2 | movie |
| 黑暗 | hak1 am3 | dark |
| 危險 | ngai4 him2 | dangerous |
| 安全 | on1 cyun4 | safe |
| 事件 | si6 gin6 | incident |
| 黑色 | hak1 sik1 | black |
| 意外 | ji3 ngoi6 | accident |
| 神 | san4 | God |
| 鬼 | gwai2 | ghost |
| 香港 | hoeng1 gong2 | Hong Kong |
| 政府 | zing3 fu2 | government |
| 政治 | zing3 zi6 | politics |
| 社會 | se5 wui6 | society |
| 中國 | zung1 gwok3 | China |
| 世界 | sai3 gaai3 | world |
| 垃圾 | laap6 saap3 | garbage |
| 共產黨 | gung6 caan2 dong2 | communist party |
| 和平 | wo4 ping4 | peace |
| 地方 | dei6 fong6 | place |
| 健康 | gin6 hong1 | health |
| 精神 | zing1 san4 | mind |
| 醫生 | ji1 saang1 | physician |
| 醫院 | ji1 jyun2 | hospital |
| 運動 | wan6 dung6 | exercise |
| 身體 | san1 tai2 | body |
| 病人 | beng6 jan4 | patient |
| 精神病 | zing1 san4 beng6 | psychaitric disorder |
| 疾病 | zat6 beng6 | illness |
| 藥物 | joek6 mat6 | medication |
| 時間 | si4 gaan3 | time |
| 金錢 | gam1 cin4 | money |
| 生命 | saang1 ming6 | life |
| 食物 | sik6 mat6 | food |
| 重要 | zung6 jiu3 | important |
| 人物 | jan4 mat6 | character |
| 珍惜 | zan1 sik1 | cherish |
| 付出 | fu6 ceot1 | contribute |
| 故事 | gu3 si6 | story |
| 美食 | mei5 sik6 | delicacy |
| 人生 | jan4 sang1 | life |
| 回憶 | wui4 jik1 | recall |
| 未來 | mei6 loi4 | future |
| 夢想 | mung6 soeng2 | dream |
| 希望 | hei1 mong6 | hope |
| 美好 | mei5 hou2 | wonderful |
| 理想 | lei5 soeng2 | ideal |
| 過去 | gwo3 heoi3 | past |
| 現實 | jin6 sat6 | reality |
| 將來 | zoeng1 loi4 | future |


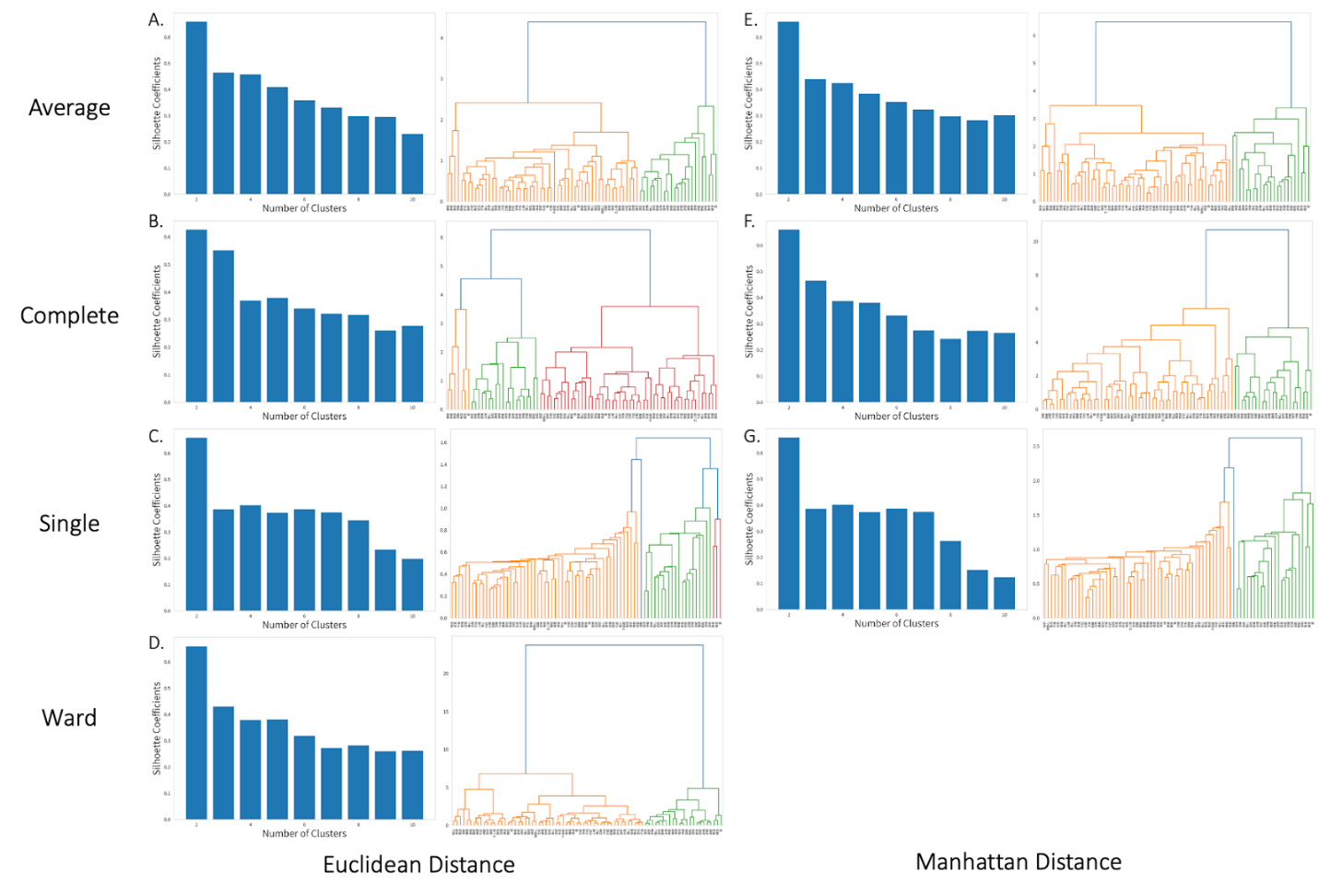


**Figure S1. Hierarchical clustering based on the original ratings of valence, arousal, dominance and concreteness by combinations of distance and linkage function.** A. Euclidean distance and average linkage function; B. Euclidean distance and complete linkage function; C. Euclidean distance and single linkage function; D. Euclidean distance and ward linkage function; E. Manhattan distance and average linkage function; F. Manhattan distance and complete linkage function; G. Manhattan distance and single linkage function.


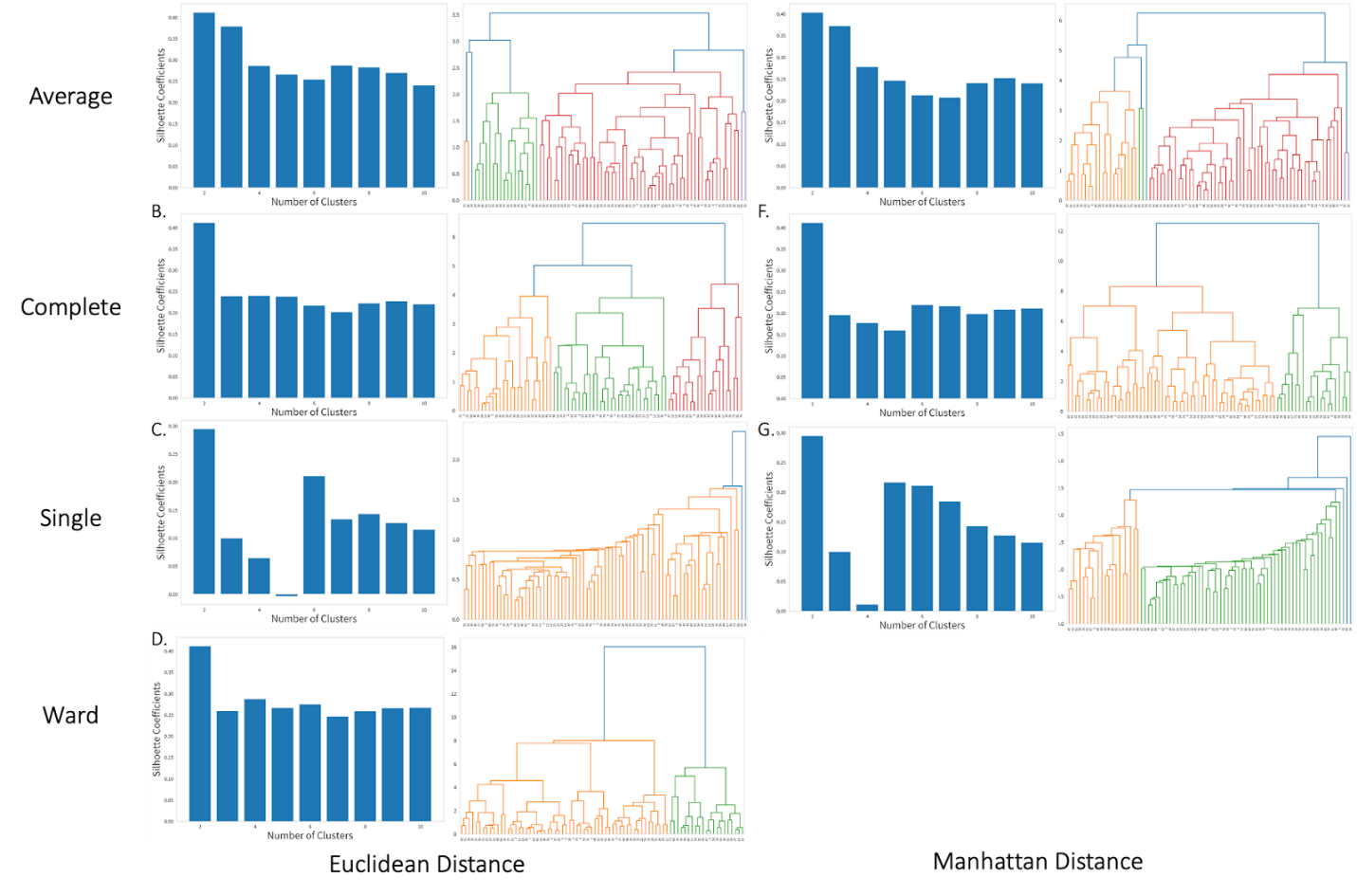


**Figure S2. Hierarchical clustering based on the standardized ratings of valence, arousal, dominance and concreteness by combinations of distance and linkage function.**  A. Euclidean distance and average linkage function; B. Euclidean distance and complete linkage function; C. Euclidean distance and single linkage function; D. Euclidean distance and ward linkage function; E. Manhattan distance and average linkage function; F. Manhattan distance and complete linkage function; G. Manhattan distance and single linkage function
